# Computational Depth of Anesthesia via Multiple Vital Signs Based on Artificial Neural Networks

**DOI:** 10.1155/2015/536863

**Published:** 2015-10-13

**Authors:** Muammar Sadrawi, Shou-Zen Fan, Maysam F. Abbod, Kuo-Kuang Jen, Jiann-Shing Shieh

**Affiliations:** ^1^Department of Mechanical Engineering and Innovation Center for Big Data and Digital Convergence, Yuan Ze University, Taoyuan, Chung-Li 32003, Taiwan; ^2^Department of Anestheology, College of Medicine, National Taiwan University, Taipei 100, Taiwan; ^3^Department of Electronic and Computer Engineering, Brunel University London, Uxbridge UB8 3PH, UK; ^4^Missile & Rocket System Research Division, National Chung-Shan Institute of Science and Technology, Taoyuan, Longtan 32500, Taiwan; ^5^Center of Biomarkers and Translational Medicine, National Central University, Chung-Li 32001, Taiwan

## Abstract

This study evaluated the depth of anesthesia (DoA) index using artificial neural networks (ANN) which is performed as the modeling technique. Totally 63-patient data is addressed, for both modeling and testing of 17 and 46 patients, respectively. The empirical mode decomposition (EMD) is utilized to purify between the electroencephalography (EEG) signal and the noise. The filtered EEG signal is subsequently extracted to achieve a sample entropy index by every 5-second signal. Then, it is combined with other mean values of vital signs, that is, electromyography (EMG), heart rate (HR), pulse, systolic blood pressure (SBP), diastolic blood pressure (DBP), and signal quality index (SQI) to evaluate the DoA index as the input. The 5 doctor scores are averaged to obtain an output index. The mean absolute error (MAE) is utilized as the performance evaluation. 10-fold cross-validation is performed in order to generalize the model. The ANN model is compared with the bispectral index (BIS). The results show that the ANN is able to produce lower MAE than BIS. For the correlation coefficient, ANN also has higher value than BIS tested on the 46-patient testing data. Sensitivity analysis and cross-validation method are applied in advance. The results state that EMG has the most effecting parameter, significantly.

## 1. Introduction

The general anesthetic drug occurs in the brain [1]. Due to that very decisive reason, it would be reasonable to monitor the brain activity by examining the EEG to assess the DoA [2]. Several studies have been addressed to evaluate the relationship between the EEG and the anesthesia [3–5]. EEG continuous signals state the complicate nonlinearity and progressive properties [6, 7] and are frequently interfered by other signals, like the electric power and elctrosurgical knives. These issues highly possibly create severe difficulty [8].

Several vital signs were used for the DoA analysis. A study concluded the power spectral analysis of heart rate variability may be a practical use for measuring DoA [9]. Horiguchi and Nishikawa evaluated studies of anesthesia based on monitoring the heart rate with the drug propofol [10, 11]. Beside the heart rate consideration, PRST (i.e., systolic blood pressure, heart rate, sweating, and tears), is also utilized as the standard measurement of the autonomic reaction in clinical practice [12]. An investigation is also conducted related to the heart rate and blood pressure to the lumbar epidural [13].

Apart from the ECG signal processing, there are several cases which were studied linking the EMG and DoA, even though a study concluded that there was no EMG effect to cerebral state monitor (CSM) or BIS index in children [14]. However, a case indicating a strong correlation between EMG and CSM in an ICU patient was conducted by Boroojeny [15]. The consciousness monitor machine, index of consciousness (IoC), was introduced by the Morpheus Medical Company. The IoC machine, estimating the anesthesia index by using the fuzzy inference algorithm, also shows the EMG bar and burst suppression ration (BSR). A study by Revuelta et al. which emphasizes the evidence of a rapid change in the IoC, accompanied by a rise of EMG, is likely due to the response of the augmented muscle activity [16]. Another commercial product, by GE Healthcare Entropy Module (formerly Datex-Ohmeda M-Entropy), provides the state entropy (SE), from frequency range of 0.8 Hz to 32 Hz, and response entropy (RE), including the facial EMG, has frequency range from 0.8 Hz to 47 Hz. For this machine, the EMG is evaluated as a signal rather than an artifact [17].

In some cases, when the surgery does not require full general anesthesia, some sedative drugs are utilized to reduce the cognitive activity [19]. Having the previously stated considerations, it is highly probable that EEG signal is considered in addition to other signals that are related to the cardiovascular system such as muscle movement and other measures investigated by previous studies to assist the medical doctor to interpret the DoA. Therefore, this study aims at investigating the DoA system based on collection of signals such as sample entropy of the continuous EEG signal, mean values of heart rate, both systolic and diastolic blood pressure, pulse, signal quality index (SQI), and EMG. This study also evaluates the sensitivity analysis in order to investigate the partial effect by the inputs to the output.

## 2. Materials and Methods

**Figure 1:**
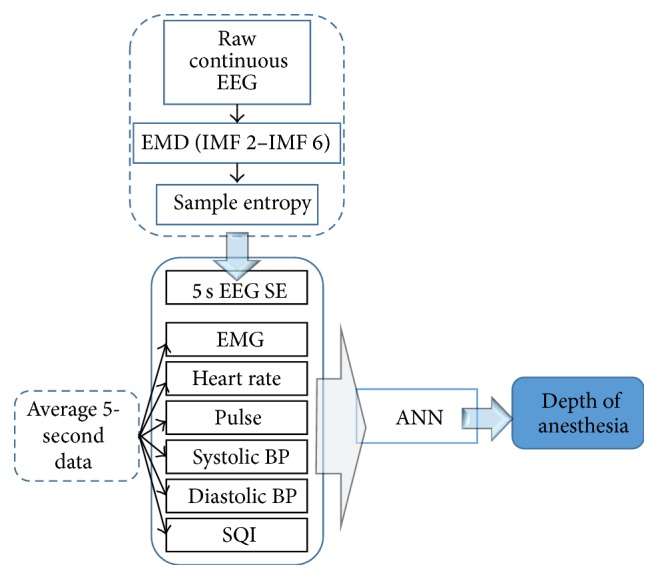
Depth of anesthesia modeling flowchart.

This research is approved by Institutional Review Board (IRB) and written informed consent was obtained for the permission by the patients. In this study, the data was collected from the patients in surgical operation room at the National Taiwan University Hospital (NTUH) in Taipei, Taiwan. The total 63-patient data were analyzed. All of the patients had general anesthesia. Physiological monitor equipment, Phillips IntelliVue MP60 utilized by BIS Quatro Sensor module, was coupled to a laptop as a data-logging system. The logged data, for the input of the modeling, are the mean data of the heart rate, pulse, blood pressure, and signal quality index (SQI), having sampling rate 0.2 Hz. In order to evaluate the model and compared to the BIS signal, whose sampling rate is also 0.2 Hz, the raw 125 Hz EEG signal is filtered and analyzed each 5 seconds, 625 points, to have a sample entropy (SE) index. In this case, the output data was produced by 5 medical doctors who concluded the anesthesia level graphically after evaluating the vital signs. This 5-doctor output was first digitized [20] and resampled at 0.2 Hz, as well as BIS frequency and other input parameters, eventually being an averaged value. The whole system is shown in Figure 1. The data is analyzed using several algorithms coded in MATLAB language (MathWork Inc., Natick, Massachusetts, USA).

### 2.1. Empirical Mode Decomposition

**Figure 2:**
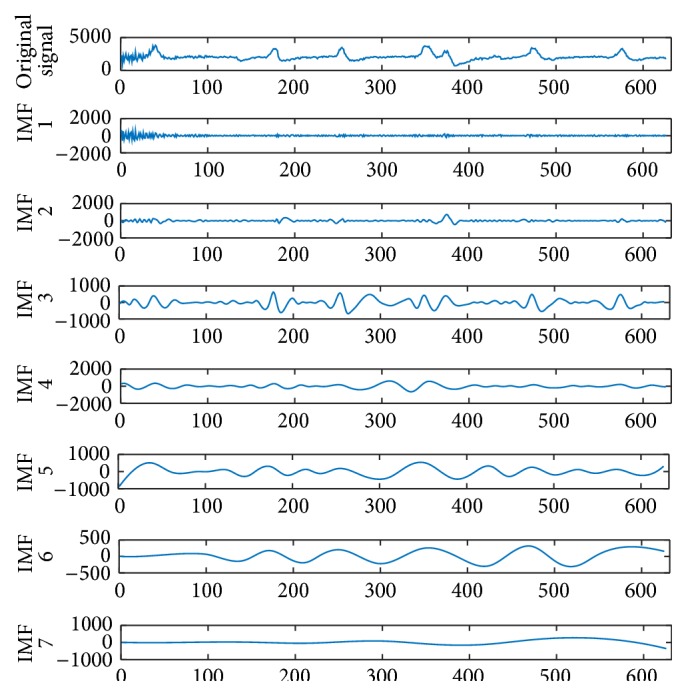
Five-second EMD-filtered EEG.

Empirical mode decomposition filtering algorithm, proposed by Huang et al., has been used for the studies related to the signal filtering problem [21, 22]. This penetrating method, in order to extract the correct information from the continuous signal, should be performed in advance. EMD working principle is by decomposing the time-series signal into a specific finite sum of the components based on the considerable frequency ranges, called intrinsic mode functions (IMFs). Consider

(1) $$\begin{array}{c} x\left(\;t\;\right)=\overset{n}{\underset{i=1}{\sum }}c_{i}\left(\;t\;\right)+r_{n}\left(\;t\;\right), \end{array}$$

where *x*(*t*) is the time domain-based original signal, *c*_*i*_(*t*) is the *i*th IMF, and *r*_*n*_(*t*) is the residual signal. Thus the appropriate signal, evaluated by the IMFs based on the frequency domain, will be merged to achieve the filtered signal. According to our previous study conducted by Huang et al., the IMF 2 to IMF 6 are the most important IMFs due to the frequency ranges appearing between 0.8 Hz and 32 Hz, which are the EEG's frequencies [23]. By Figure 2 the 5-second EMD-filtered EEG can be seen.

### 2.2. Sample Entropy

The entropy is originally known as the thermodynamics property to determine the disorder. The higher entropy means the less regular the pattern or the sequence to be recognized. There were several previous studies administered to accomplish this objective [24, 25]. Sample entropy is a coarse-grained time series calculation. It results in {*y*_1_, *y*_2_, *y*_3_,…, *y*_*N*_}. The length of the two patterns, *m*,   *X*_*m*_(*i*) = {*y*_*i*_,…, *y*_*i*+*m*−1_} and *X*_*m*_(*j*) = {*y*_*j*_,…, *y*_*j*+*m*−1_}, is assigned to calculate the number of *X*_*m*_(*j*) for the condition of

(2) dXmi,Xmj≤rr≥0,dXmi,Xmj=max⁡yi+k,yj+k,k∈0,m−1,  j∈1,N−m,  i≠j.

*B*_*i*_^*m*^(*r*) is the outcome of all *Xm*(*i*) similar to *Xm*(*j*) and the average for *i* ∈ [1, *N* − *m*] is

(3) $$\begin{array}{c} B^{m}\left(\;r\;\right)={\left(\;N-m\;\right)}^{-1}\overset{N-m}{\underset{i=1}{\sum }}B_{i}^{m}\left(\;r\;\right). \end{array}$$

When the length increases to *m* + 1,

(4) $$\begin{array}{c} A^{m}\left(\;r\;\right)={\left(\;N-m\;\right)}^{-1}\overset{N-m}{\underset{i=1}{\sum }}A_{i}^{m}\left(\;r\;\right). \end{array}$$

Finally the function of sample entropy can be calculated as follows:

(5) $$\begin{array}{c} S_{E}\left(\;m,r,N\;\right)=-\ln\left(\;\frac{A^{m}\left(r\right)}{B^{m}\left(r\right)}\;\right), \end{array}$$

where *m* is the space dimension, *r* is standard deviation, and *N* is the length of the time series. This study uses the parameters of *m* and *r* which are 2 and 0.15, respectively, according to the previous study by Costa et al. [26].

### 2.3. Artificial Neural Networks

**Figure 3:**
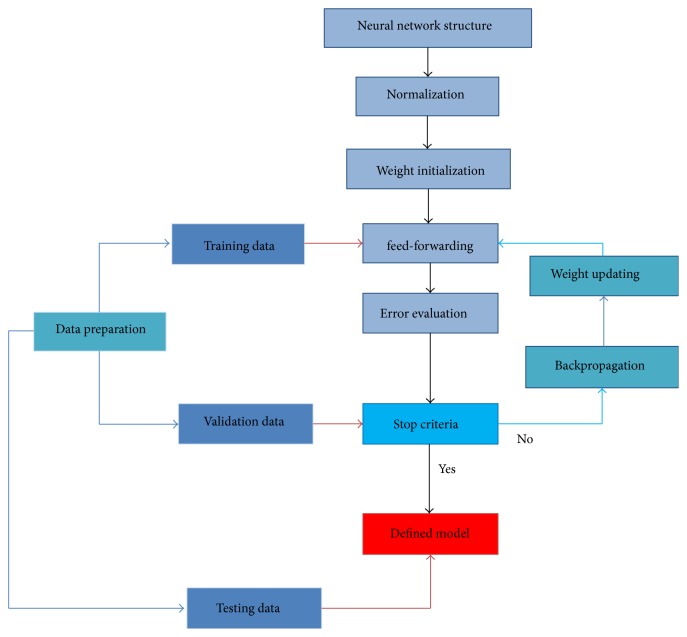
Artificial neural network flowchart.

Artificial neural network is a structure developed particularly to imitate the human thinking. Enormous highly interconnected processing elements operating parallel work for the network. This steers ANN to be used in many areas [27]. The neural network is trained to learn some patterns of the input-output modeling system. In training, a backpropagation neural network (BPNN) is one of the most well-known methods working by evaluating the error model backwardly.  Figure 3 shows how the BPPNN works, starting from structure of the system, normalization, weight initialization, feed-forwarding, computation of the error, backpropagation, updating the weights, and testing the fixed model.

For data preparation, all the alphabetical data should be altered to numerical value. Normalizing data should be performed for range from 0 to 1 due to the nature of the log sigmoid transfer function used in the model. All normalized data and weights are included in the feed-forward step to be evaluated by the log sigmoid system.

### 2.4. Sensitivity Analysis

In order to evaluate the behavior of the inputs and the outputs, sensitivity analysis is the appropriate consideration [28]. The partial derivative of the networks' input for the output of the sensitivity analysis is utilized in this study by leave-one out method. The following algorithms are as follows:

- First of all, normalize all the input and output corresponding to their own specific parameters, zero as the minimum value and one as the maximum value.
- Average all the input variables and simulate them to get the output as the target of the comparison value.
- Sequentially and partially, change each input from 0 to 1, by 0.1 increments, and others keep being constant to examine the mean-squared error (MSE) of the actual output and target differences and analyze how sensitive the variable for the system.
- Lastly, make the ranking of each input variables based on the error produced by the model. The more the MSE, the more sensitive the input for the model.

## 3. Results

In this study, backpropagation artificial neural network is utilized. The single hidden layer, 10 hidden nodes, 10,000 epochs, small learning rate of 0.005, and 0.15 of momentum term are applied to model the depth of anesthesia. This relatively low learning rate is compensated by the enormous epoch. In order to get the precise model, computational time consideration, due to the epoch number, is ignored. Totally 63-patient data is addressed, for both modeling and testing, 17 and 46 patients, respectively. In order to evaluate more details about the relationship between the inputs and the output variable, the 10-fold cross-validation and sensitivity analysis are performed. The averaged result from each fold will create a single model to determine the DoA. This method is also used to evaluate the sensitivity analysis by averaging the errors from each fold to decide the rank of the parameter affecting the output.

**Figure 4:**
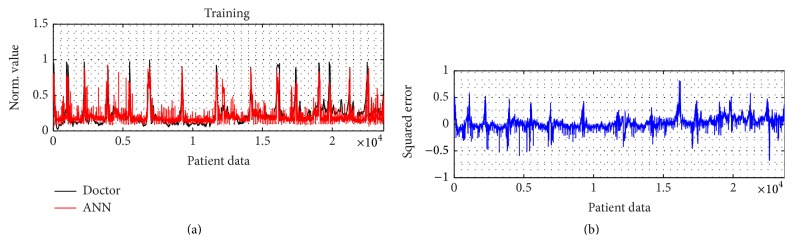
ANN training result. (a) ANN training performance compared to the doctor decision and (b) the squared-error between the model and the doctor's consciousness index of 16 patients.

**Figure 5:**
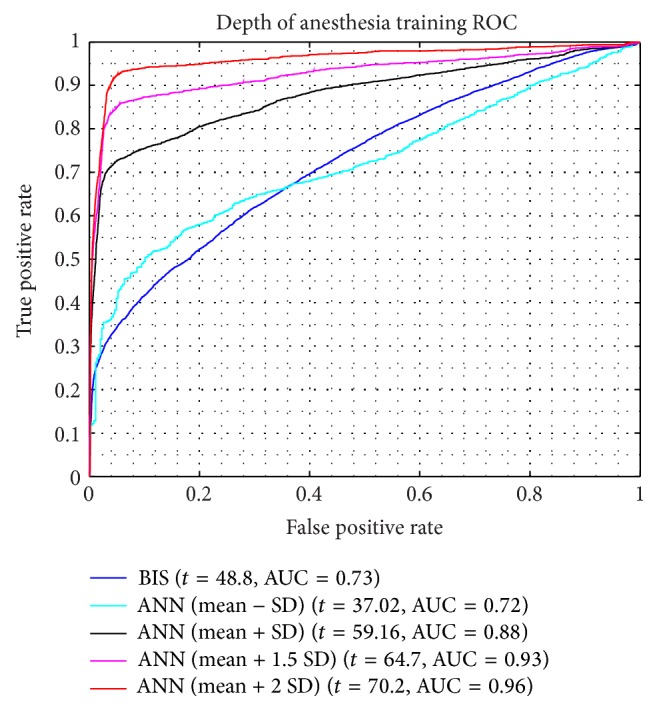
The training ROC curve and AUC results by several thresholds [*t* = threshold, AUC = area under the curve].

For the training result, how the ANN model in approaching the doctor's index and its MSE are shown in Figures 4(a) and 4(b). The model is relatively better in dealing with the unconscious levels, indicated by the lower error, than facing the conscious stage. For its performance and validation model result, it can be seen by Figures 9 and 10, respectively. In order to calculate the ROC curve and its AUC, as shown in Figure 5, the threshold between conscious and unconscious has to be decided. By referring to a previous study by Gajraj et al. [18], 48.8 is defined as the BIS mean value for the consciousness level, in range of 1 to 94, while the conscious mean value is 89.5, by the lowest conscious value which is 70 and the highest which is 97.

In this study, several thresholds are defined based on the mean and the standard deviation of the training data. The combination of those two parameters, started by the lowest threshold value, 37.02, producing AUC is 0.72, and the highest threshold value, 70.2, achieving 0.96 of AUC. In this case, the sum of the mean and double standard deviation, which is the highest threshold in order to distinguish between conscious and unconscious, is relatively similar to the lowest conscious value defined by Gajraj et al.; it has 0.2% error exceeding their study's thresholding value.

**Figure 6:**
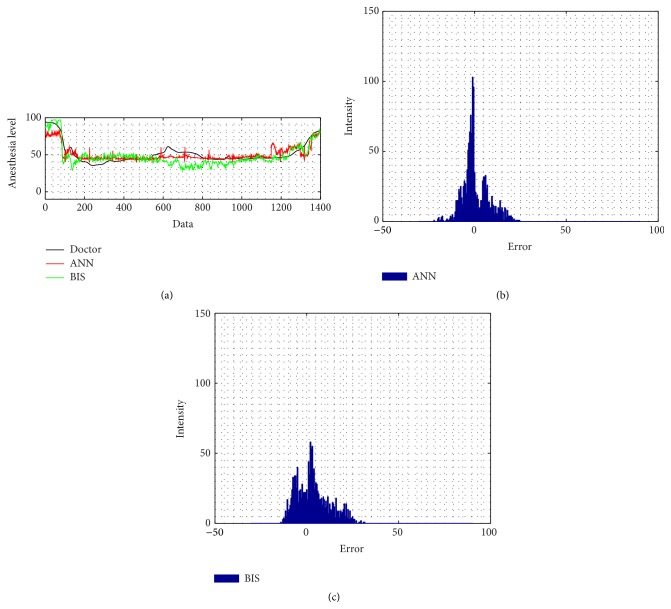
A clean patient signal: (a) BIS signal and ANN performance in comparison with doctor's index; (b) ANN error; (c) BIS error.

**Figure 7:**
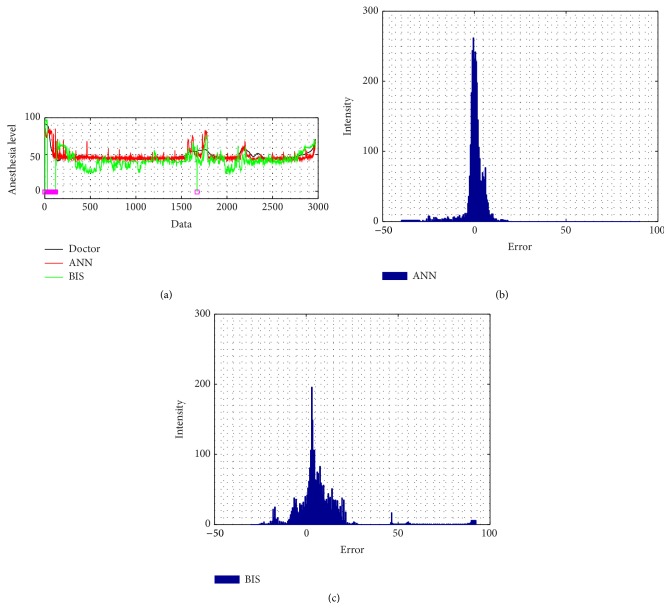
A noisy patient signal: (a) BIS signal and ANN performance in comparison with doctor's index; (b) ANN error; (c) BIS error (magenta squares indicate the lost signal).

The intensity of 46-case testing ANN absolute error is more closely distributed to zero than the BIS' error. The correlation coefficient of the ANN is also better than BIS result, by evaluating its distribution; ANN has 0.66 ± 0.21; meanwhile BIS has 0.48 ± 0.36, shown by Figure 11. For the noise problem, Figures 6 and 7 reveal how the ANN and BIS deal with the clean and noisy signal.  Figure 7 in particular shows that, for conditions in noisy environment, the BIS signal has noise contamination due to the electrosurgical knife which cause the signal drops to negative values, marked by the magenta squares. However, in this case, the ANN model provides a more stable and noise-free behavior.

**Figure 8:**
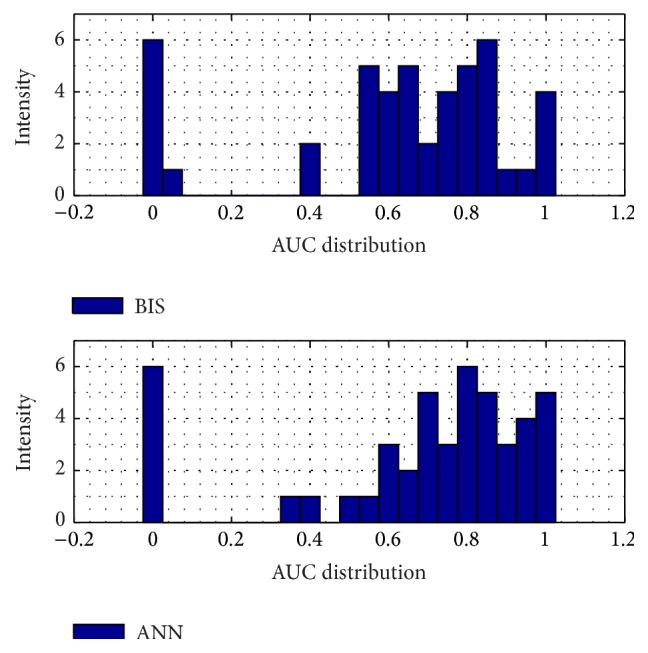
Testing model ROC area under the curve of BIS and ANN, with the mean BIS-fixed threshold [18].

**Figure 9:**
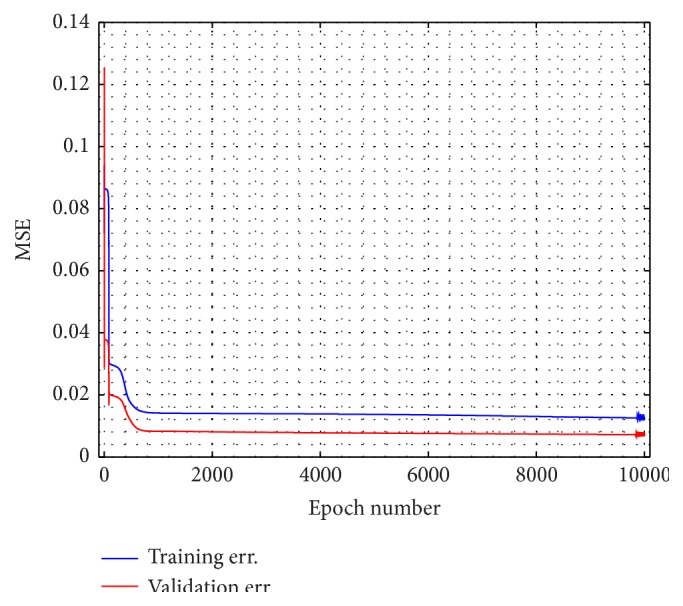
Modeling performance error curve.

**Figure 10:**
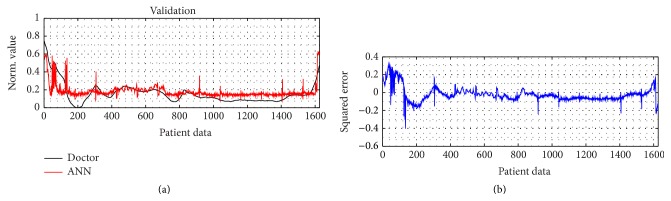
ANN validation result. (a) ANN validation performance compared to the doctor decision and (b) the squared error between the model and the doctor's consciousness index of a patient.

**Figure 11:**
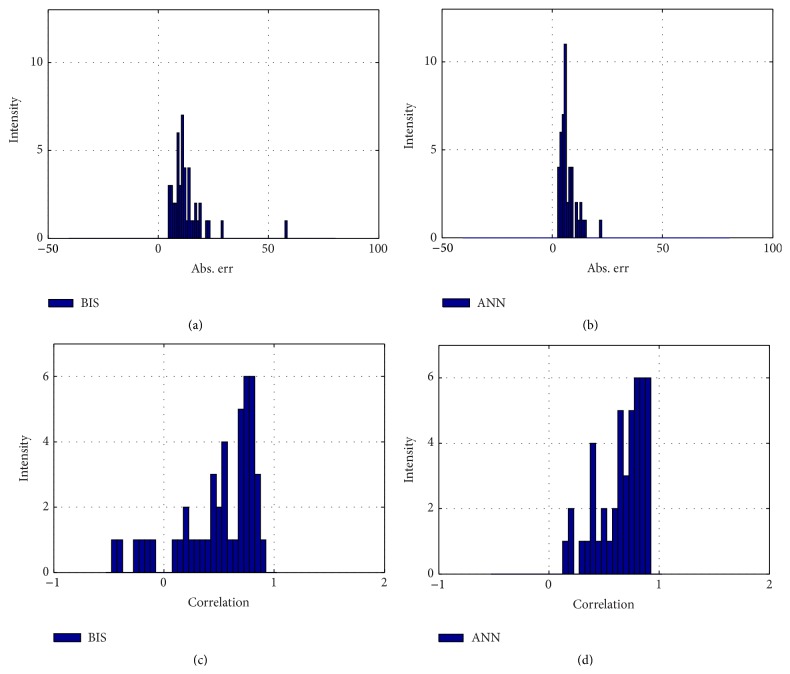
Testing evaluation. (a) The absolute error of BIS. (b) The absolute error of ANN. (c) The correlation coefficient of BIS. (d) The correlation coefficient of ANN. All is compared to the doctor's index.

**Figure 12:**
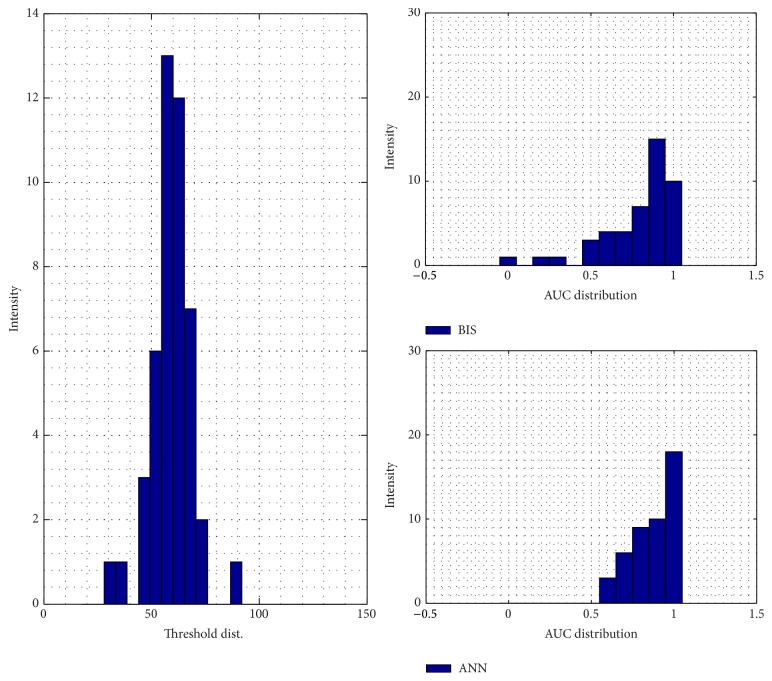
Testing model AUC distribution by the sum of mean and standard deviation consciousness threshold.

**Figure 13 fig13:**
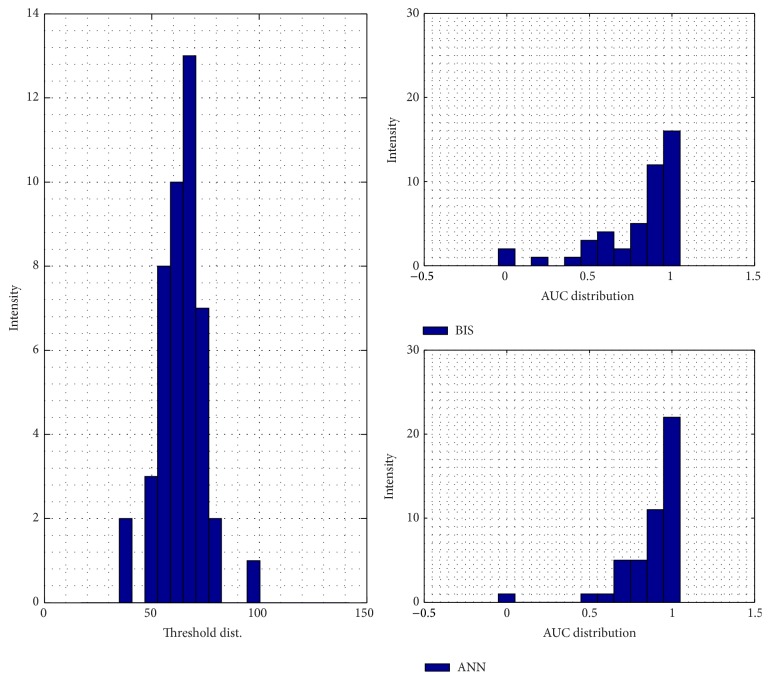
Testing model AUC distribution by the sum of mean and 1.5 of standard deviation consciousness threshold.

**Figure 14:**
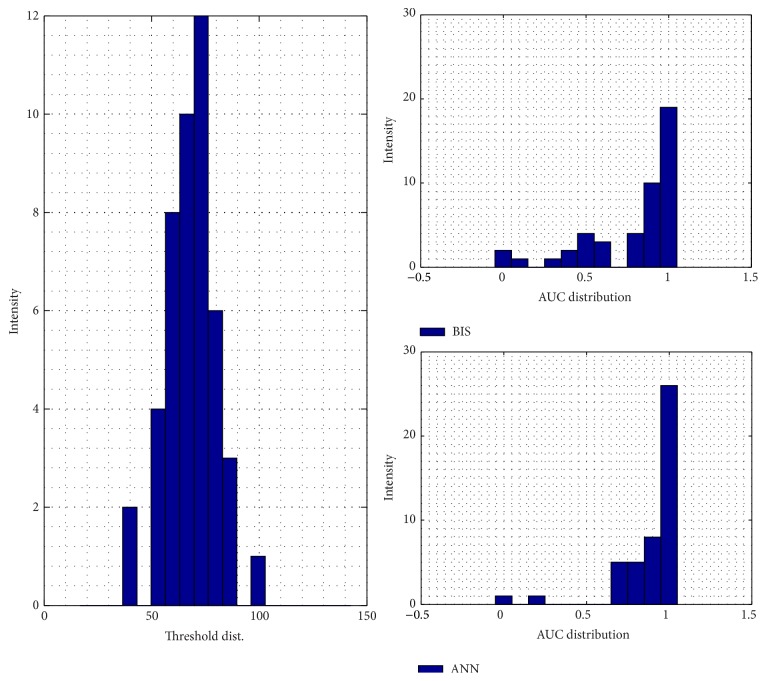
Testing model AUC distribution by the sum of mean and 2 of standard deviation consciousness threshold.

Another way to evaluate the performance of the testing models is by calculating the area under the curve (AUC) of the receiver operating characteristics (ROC).  Figure 8 shows that the threshold is fixed by 48.8 by using Gajraj study's reference. The threshold procedure is taken by finding each mean testing patient data and added with its various standard deviations. Higher threshold will produce bigger AUC of the ROC curve. The figures for the several thresholds can be seen from Figures 12 [Fig fig13]–14.

In purpose of evaluating the generalization the data distribution between the modeling data and the testing data, the 10-fold cross-validation is performed. Each single training and validation data is switched; then the modeling stage starts to train the new model. This model will evaluate the fixed 46-case testing data. This algorithm is also applied to evaluate how general the model in interpreting the testing by utilizing the sensitivity analysis.

For the total 46 patients, the cross-validation method utilized to evaluate the distribution of the data can be seen by the standard deviation in Table 1. First each fold evaluates the testing data in order to produce MAE and standard deviation. The total MAE only are then averaged to form a total system mean and the standard deviation, marked by “*∗*.” By having this result, that is, 6.61 ± 0.15, the data is relatively similar to analyze these 46 patients. In order to make the model more robust, the ensemble system, based on several studies [29, 30], has been addressed to the whole folds, producing MAE of 6.54 with 6.69 of standard deviation, before concluding the index. BIS has 12.31 of MAE and 13.06 of standard deviation, meaning that the ANN has better ability to predict the DoA than BIS.

**Figure 15:**
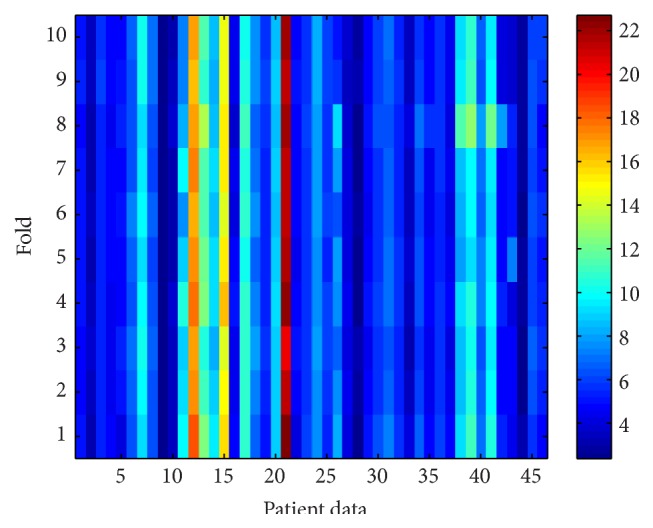
Each single fold performance result; color-bar shows the mean error (the lower the better the model).

**Figure 16:**
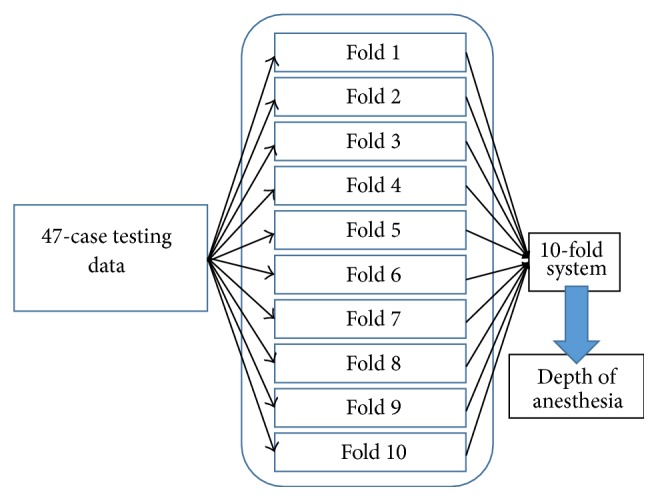
Cross-validation mechanism.

**Figure 17:**
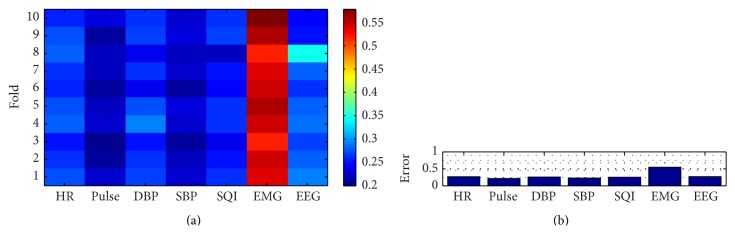
10-fold cross-validation parameter sensitivity analysis result. (a) The single fold mean-squared error for each parameter. (b) The total fold parameter average errors.

The sensitivity evaluation is eventually utilized to evaluate the ranking of the input variables, in order to investigate the relationship to the output. Here, the 10-fold cross-validation evaluates the error from each variable into every single fold; it can be seen by Figures 15 and 16. The error is then averaged to evaluate the parameters, identically to the previous mechanism, shown by Figure 17. It concludes that the EMG signal has the highest influence followed by EEG, heart rate, mean diastolic blood pressure, signal quality index, mean systolic blood pressure, and pulse. The EMG, which has very confident index which is in the first rank, has significant difference compared to the second rank, EEG (*P* value < 0.05). However, for the following rankings, EEG, heart rate, mean diastolic blood pressure, and SQI, second to fourth, are not significantly different (*P* value > 0.05). For the fifth, mean systolic blood pressure has significant different with the SQI, that is, in the fourth position. Pulse has the less influence to the depth of anesthesia, even though it does not have rapid difference with the systolic blood pressure.

## 4. Discussion and Conclusions

Deciding the index of the anesthesia consciousness in surgical procedure is extremely critical. In practical, a number of parameters should be considered. On the other hand, in the operating theater while performing and evaluating the anesthesia consciousness index, noise is highly likely to interfere with the decisive signal enlightening the index. The classification method should be able to precisely recognize the patients being either awake or sleep.

The EEG signal is decomposed by EMD method and recomposed by the frequency of 0.8 Hz to 32 Hz, the IMF 2 to IMF 6, to purify from the noise. This filtered signal is then extracted to evaluate the sample entropy index. This signal is resampled at 0.2 Hz in order for the BIS sampling frequency. The sample entropy of the EEG is then combined with every 5-second mean signal, EMG, heart rate, pulse, systolic and diastolic blood pressure, and signal quality index to evaluate the DoA index as the input and the doctor index as the output of the ANN modeling. The receiver operating characteristics (ROC) curve and the 10-fold cross-validation are performed in advance to evaluate the model and for the sensitivity analysis.

There are some perspectives for considering or filtering the muscle activity. The EMG signal is commonly classified as the artifact effect for the EEG data logging cases due to the muscle activities [31–33]. However, in another study by Viertiö-Oja et al. [17], the EMG is decided as a signal, instead of an artifact. In advance, Boroojeny showed a case which marks a strong correlation between EMG and CSM in an ICU patient [15] and a study by Revuelta et al. highlights the significant change in the IoC, by a rise of EMG which is possibly due to the feedback of the enhanced muscle activity [16].

By this study, totally 63-patient data is addressed, for both modeling and testing, 17 and 46 patients, respectively. In order to evaluate patients being either awake or sleep, the threshold is decided by a study conducted by Gajraj et al. [18]. The noisy result by the ANN will often affect the AUC of ROC curve result due to the threshold level. However when facing the noise environment in the operation room, the ANN results still provide more robust results compared to the BIS. The evaluation results also show that the ANN is better than BIS in dealing with anaesthesia by most of the cases. For parameter evaluation, sensitivity analysis is performed. The EMG is the most affecting parameters followed by EEG, heart rate, diastolic blood pressure, SQI, systolic blood pressure, and pulse.

This study is relatively novel and successful in evaluating the consciousness level to overcome some surgical procedures utilizing some drugs subsequently diminishing the effect of the EEG signal, commonly utilized as a parameter for the depth of anesthesia (DoA). Furthermore, this study provides information on the muscle activity, EMG, that in some cases are considered as the noise, significantly affecting the result to characterize the consciousness level. The results are supported by previous studies conducted by Boroojeny [15] and Viertiö-Oja et al. [17].

## Figures and Tables

**Table 1:** 46-patient testing result from each fold.

Fold	MAE ± SD
1	6.45 ± 6.61
2	6.6 ± 6.68
3	6.56 ± 6.59
4	6.79 ± 6.89
5	6.64 ± 6.75
6	6.51 ± 6.68
7	6.58 ± 6.70
8	6.94 ± 6.69
9	6.59 ± 6.69
10	6.5 ± 6.8
Mean ± SD	**6.61 ± 0.15** ^*∗*^

Note: “*∗*” means average and standard deviation of MAE.

## Appendix

The performance result of the modeling can be seen in Figure 9 showing the performance of the model, both the training and the validation data. On this picture it can be seen that the error converged in the early epoch. The model converging error becomes saturated and slightly changes after the around thousand epochs. A ten-thousand-epoch model is decided as the stopping criterion. The validation model result and the error also can be seen in Figures 10(a) and 10(b). The 46-case testing data of absolute error and the correlation coefficient comparing BIS and ANN with the doctor's index is shown in Figure 11. Several defined thresholds to evaluate the performance of the models are shown by Figures 12–14. The information about the marginal error of each single fold to the patient testing dataset based on the color map can be seen in Figure 15. The color-map distributes from the blue-based color to the red-based color. The closer the matrix color to the red-based color is, the higher the error is generated.

To form the single cross-validation model, each index created by each fold, for totally 10-fold, is then averaged, shown in Figure 16. These models have different training and the validation dataset. This algorithm also aims at producing the result of the independent testing data more generally. It works by considering the whole folds, which were trained, before averaging them into an index.

Figure 17(a) shows the error response when each parameter partially changes to each single fold. The higher error means the more sensitive the parameter to the model.  Figure 17(b) shows the averaged error of the total folds.
